# Community engagement in mobile and hard-to-reach populations: a community-based intervention for malaria elimination in a tri-national region of the Guiana Shield

**DOI:** 10.3389/fpubh.2024.1377966

**Published:** 2024-09-10

**Authors:** Irene Jimeno-Maroto, Muriel Suzanne Galindo, Jane Bordalo Miller, Yann Lambert, Carlotta Carboni, Teddy Bardon, Lorraine Plessis, Stephen Vreden, Martha Suarez-Mutis, Maylis Douine, Alice Sanna

**Affiliations:** ^1^CIC INSERM 1424, Cayenne Hospital, Cayenne, French Guiana; ^2^Development, Prevention, Monitoring and Border Cooperation (DPAC-Fronteira), Oiapoque, Brazil; ^3^Foundation for the Advancement of Scientific Research in Suriname (SWOS), Paramaribo, Suriname; ^4^Oswaldo Cruz Institute, Oswaldo Cruz Foundation (Fiocruz), Rio de Janeiro, Brazil

**Keywords:** community-based interventions, community engagement, IEC, malaria elimination, Guiana Shield, mobile and hard-to-reach populations

## Abstract

Several countries of the Guiana Shield are aiming at the control and elimination of malaria in areas where Artisanal and Small-scale Gold Mining (ASGM) activities predominate, raising questions about how to strengthen community engagement to improve the effectiveness of health programs. The Curema project focuses its intervention on the mobile and hard-to-reach ASGM population, complementing the efforts of national programs in the Guiana Shield. The Curema intervention combines targeted drug administration for suspected *Plasmodium vivax* asymptomatic carriers, the Malakit distribution, and health education activities. The primary goals of this manuscript are to outline a pathway to foster community participation in the Curema project aimed at eliminating malaria. Thus, it presents a vision of the challenges that the AGSM community poses in terms of community participation for an asymptomatic problem; and highlights the community-based model and the Information, Education and Communication (IEC) components as foundations for participation. In addition, it also presents culturally sensitive IEC strategies designed through iterative and collaborative consultative processes and other bottom-up outreach activities. The community engagement approach facilitates adaptability and responsiveness in a complex, evolving context increasing the effectiveness of interventions.

## Background

In the Guiana Shield—an ecoregion that encompasses Guyana, Suriname, French Guiana, and some parts of Venezuela, Colombia, and Brazil—malaria transmission is mainly concentrated in areas with Artisanal and Small-scale Gold Mining (ASGM), involving a highly mobile and transboundary population (1). In the context of their remote and often informal or illegal activity, ASGM related individuals often struggle to access healthcare services, and therefore resort to self-medication with black market drugs or the use of traditional or popular medicine (1–4). The Americas reported 0.55 million malaria cases in 2022, with Venezuela, Brazil, and Colombia contributing over 73% (5). Despite this endemic context, one part of the Guiana Shield region, composed by Suriname, French Guiana (France) and the Amapá State (Brazil), made huge progress in malaria control over the ten last years. In 2022, the reported indigenous cases were: 0 in Suriname, 21 in French Guiana (France), and 2802 in Amapá (Brazil) (5, 6) compared to 795, 1,209, and 19,002 cases respectively in 2011. Suriname is actively engaged in the World Health Organization (WHO) Global Malaria Program's E-2025 initiative since 2017, joined by French Guiana in 2021 (5). Nevertheless, given the mobility patterns of the ASGM population across the Amazon region, sustaining joint multinational efforts for malaria control and elimination remains crucial (7).

While healthcare for malaria is provided for free in Suriname, France, and Brazil, border transmission remains problematic (8, 9). As residual malaria in the region is linked to gold mining (1), Suriname take direct initiatives for prevention and active case detection within informal, unauthorized and remote settlements by the National Malaria Elimination Program, through a network of Malaria Service Deliverers and private-sector primary care facilities (8). The Brazilian State of Amapá and its municipalities, as part of the decentralized national health system, executes management and vector control preventive activities to manage inland reported cases and outbreaks, in accordance with the recommendations of the National Malaria Program. In French Guiana the case management and preventive activities are also provided for free to this public and are embedded horizontally in the health care system; in the absence of a French national program for malaria elimination, the Regional Health Agency and its field partners defined in 2015 a Malaria Regional Plan to guide local control efforts. On the other hand, French Guiana employs coercive measures against illegal gold mining activity, which is a politically sensitive issue (10), and consequently, faces several constraints for health professionals to reach these remote and informally inhabited areas (11, 12). Hence, the region grapples with the complexities of the ASGM mobile, hard-to-reach, and sometimes hidden population: mobility, legal and migratory issues, environmental impacts, situations of social vulnerabilities, and health challenges (7, 10, 13–16).

The lack of access to health care frequently leads in this community delayed management of malaria symptoms and to inadequate self-treatment realized with smuggled drugs, without prior testing and often with incomplete posology. To address these challenges, the Malakit project (2018–2020) demonstrated successful international collaboration, distributing self-test and self-treatment kits (the Malakits) associated with user training, and providing malaria education to individuals involved in ASGM (2, 13, 17, 18). The kits contain 3 malaria rapid tests, an artemether-lumefantrine full course, a single low dose of primaquine and a blister of paracetamol for symptomatic treatment of fever and pain. The quasi-experimental evaluation of the Malakit strategy proved to be safe, well-accepted by the community, to led to a significant improvement in malaria symptoms management by community members. It also showed through mathematical modeling that accelerated the decline of malaria incidence in the region by 42.9% between 2018 and 2020 (4). These research results led to the scaling-up in Suriname's Malaria Program (19).

Following Malakit intervention research, the Curema project (2022–2025) addresses the emergence of *Plasmodium vivax (P. vivax)* as a dominant malaria strain (around 80% in the region) which poses hard challenges mainly due to *P. vivax* hypnozoites causing relapses, requiring a specific treatment regimen for its radical cure (5, 6, 20, 21). Curema combines three actions: (i) aa targeted drug administration (TDA) against *P. vivax*; (ii) the Malakit delivery and user training with improved education tools; (iii) and health education activities to eliminate malaria (22).

The TDA consists in providing a full *P. vivax* treatment (3 days course of chloroquine, associated with a 7 day course of primaquine or a single dose of tafenoquine) to adult asymptomatic individuals suspected of carrying *P. vivax* hypnozoites, based on specific eligibility criteria related to the participant's medical history. To explore contraindications to this treatment, prior Glucose-6-Phosphate Dehydrogenase (G6PD) deficiency screening and pregnancy test are performed, as well as questions about previous adverse drug reactions and other clinical data. To maximize the safety of the individuals treated with a radical treatment a commitment to a 14 day follow-up is mandatory through either in-person visits, phone calls, or a mobile application (APP) (22).

Top-down health interventions, especially concerning low transmission settings and/or targeting hypnozoites carriers-asymptomatic individuals-, might struggle to achieve effective participation from the community when the health initiative does not meet their interests or priorities (23, 24). The Curema project recognized the importance of community engagement is essential to overcome potential barriers in participation. Indeed, promoting community participation is a critical concern not only for regions around the world striving for malaria elimination, but also for community health interventions, in favor of the wellbeing of societies (23–27).

The participation of the community in health interventions should not be understood only as the passive reception of services, but the integration of community's perspectives, needs, and aspirations into planning, execution, and evaluation. Therefore, the core objective of the community engagement is to establish trust relationships, foster dialogue, and build partnerships. This approach recognizes the community's inherent knowledge of its unique context, suggesting that their involvement can yield effective and sustainable outcomes (24, 27, 28).

Community engagement is frequently conceptualized as a continuum of participation, ranging from minimal or symbolic involvement to substantive and meaningful collaboration (25, 26, 28–30). The progression along this spectrum comprises phases commonly referred by the literature to as: informing; consulting; collaborating; participating; and community leading. This continuum acknowledges the dynamic nature of engagement, adaptable to different circumstances and settings (25, 26, 28–30). Community-based models are found at the base of the continuum of participation and relies on a collaborative approach with non-academic partners in the process of creating knowledge to improve the understanding of a given phenomenon and the social and cultural dynamics of the community, and integrating the knowledge gained with action to improve the health (31). The community-based model underpins the Malakit and Curema projects.

The purpose of this manuscript is to outline the pathway for community engagement adopted in the Curema project: it describes the fundamental components of the Curema community-based model, the Information, Education, and Communication (IEC) strategy and materials created, and the opportunities of the bottom-up outreach activities. It also aims to present the experience accumulated during the development of this project to the international community, so that it can provide food for thought for decision-makers, activists and researchers involved in similar challenges.

## The ASGM community and the Curema intervention locations

The intervention primarily centers on the ASGM community. The term “community” denotes a cohesive group sharing common cultural, or social characteristics, common perspectives, united by shared goals in geographical locations or settings. Even if, from the external perspective, it may appear as a single community, it consists of multiple sub-groups or distinct communities of identity (32). In French Guiana, over 95% of individuals working in mining sites are of Brazilian origin, although diverse origins exist. However, Brazilian Portuguese remains the predominant and common language (10, 14, 33). Women constitute 15–30% of the ASGM population, primarily working in the mining services sector. Gold mining sites are dispersed across the region's rainforest, forming networks that connect extraction sites with support settlements, located in border areas and regional capitals. Informal social networks provide support for daily life and work, yet they lack sufficient structure to establish strong connections with public bodies. ASGM community members exhibit high geographic mobility, usually spending 2–3 months in mining camps with temporary stays at logistical sites for various purposes such as acquiring supplies, rest, medical care, visiting family, and selling or shipping gold (10, 14, 33).

**Figure 1:**
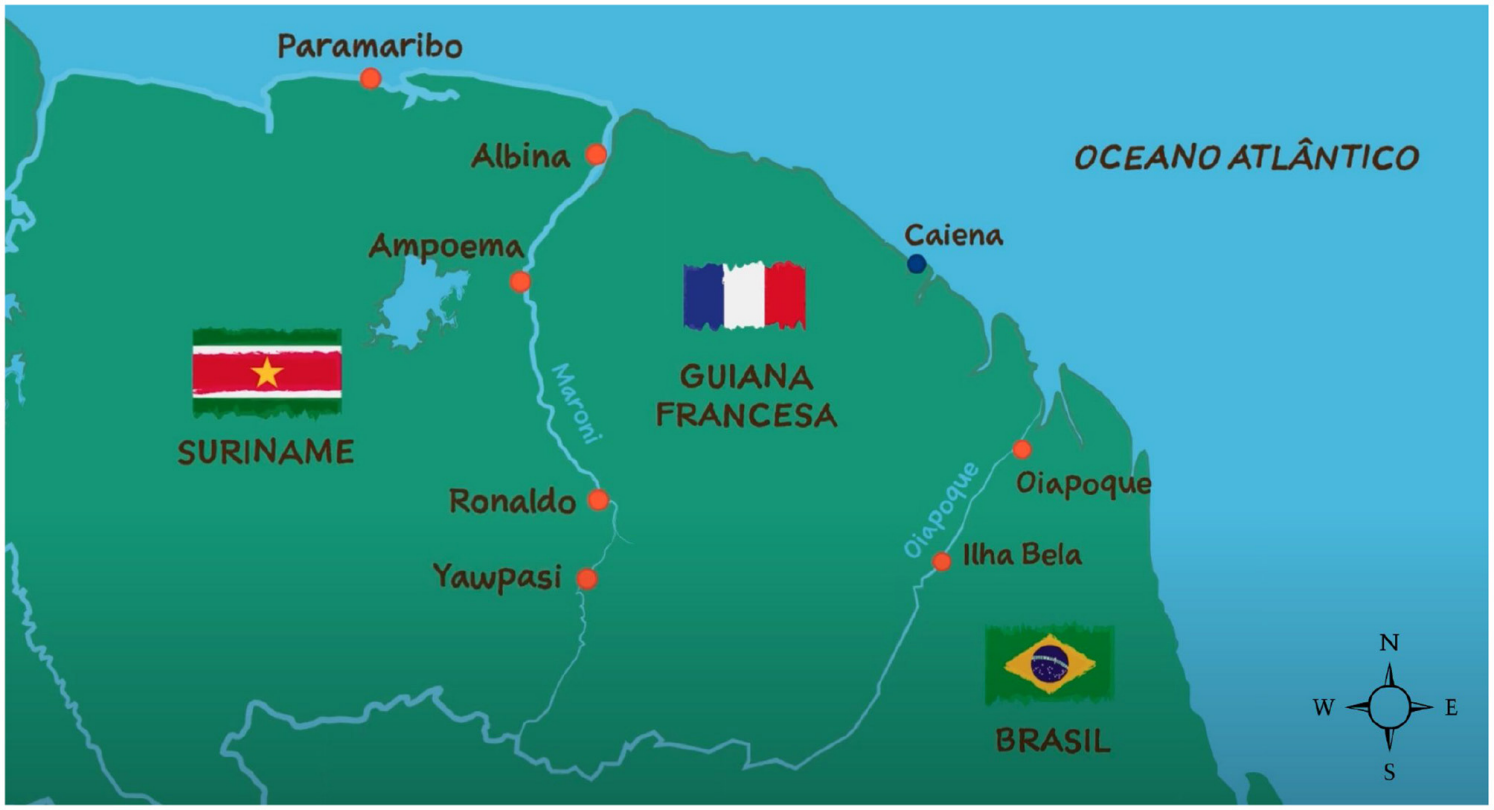
Map of the Guiana Shield illustrating the inclusion sites for the Curema project intervention. Self-produced figure.

Curema project focuses its intervention on logistical sites serving the ASGM community along the French Guiana borders instead of direct entry to gold mining sites. Seven bases are strategically established: three in cities (Oiapoque, Albina, Paramaribo) and four in informal inland settlements (Ilha Bela, Ampoema, Ronaldo, Yawpassi) (Figure 1). These sites are neutral spaces where members of the community are easy to meet and approach, as there they are not in a condition of risk of police pursuit linked to their activities or migratory status.

## The community-based Curema design

**Figure 2:**
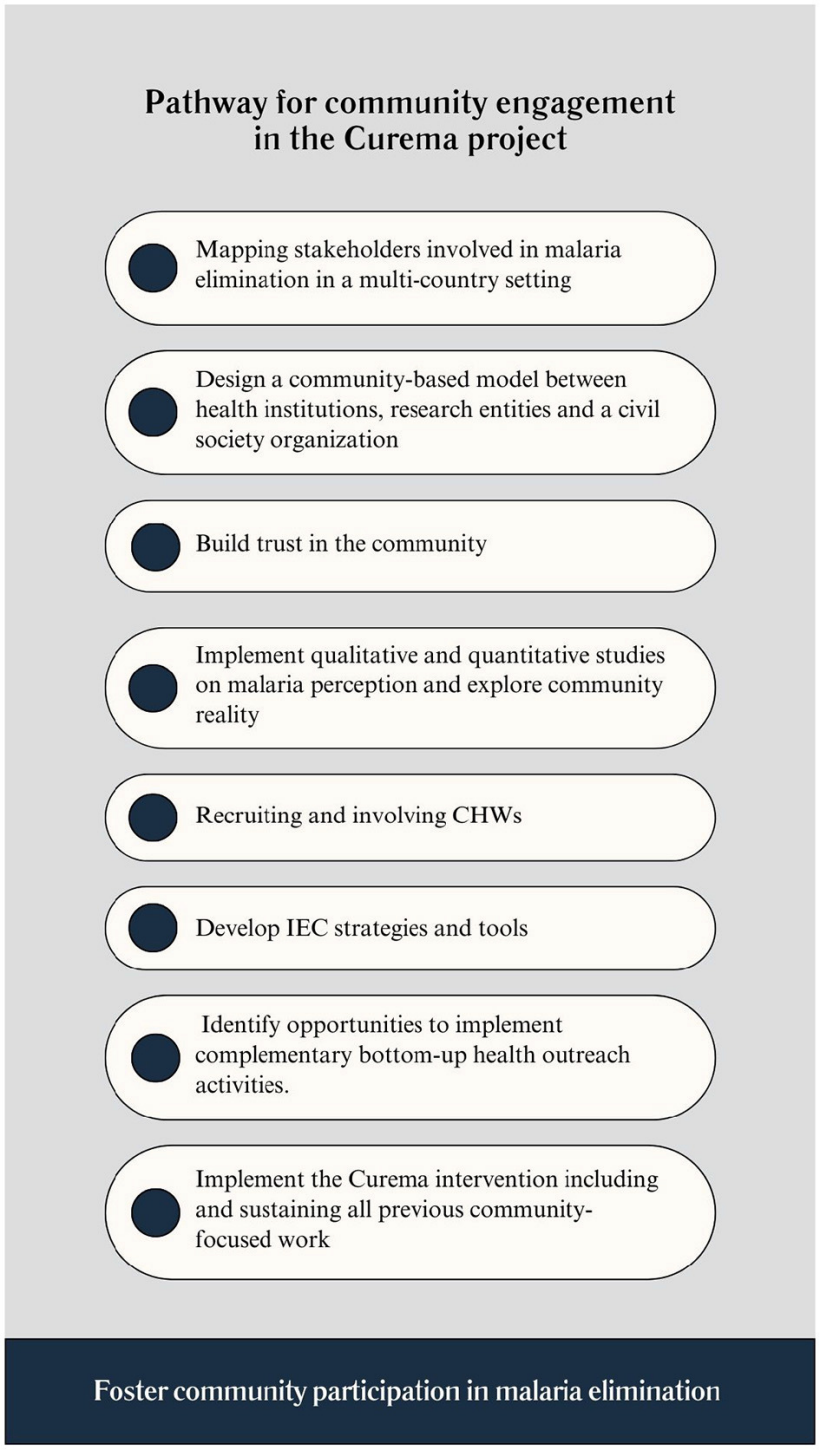
Pathway for community engagement in the Curema project.

The Curema community-based interventional research comprises several crucial elements: (a) multi-actor partnership; (b) Pre-post intervention studies and monitoring; (c) On-site visits and trust building; (d) Recruitment and involvement of community members as field workers; (e) Participatory development of the IEC (Information, Education, and Communication) tools and of study components. Figure 2 represents the path to foster community participation in the Curema project objective, which is further described below.

## A multi-actor partnership

This initiative involves a diverse array of stakeholders-jointly formulating, executing, and assessing the research initiative-, including the Cayenne Hospital Center (CHC), the Foundation for Scientific Research in Suriname (SWOS), the Suriname Malaria Program (SMP), the Oswaldo Cruz Foundation of Rio de Janeiro (Fiocruz), and the local civil society organization Association for Development, Accompaniment, Animation, and Cooperation (DPAC Fronteira). Financial support comes from the European Union, the Surinamese Ministry of Health, the Brazilian Ministry of Health, the CHC, the French Guiana Regional Health Agency, and contributions from Tropical Diseases Research-World Health Organization (TDR-WHO). This multitude and diversity of actors allows for a comprehensive approach encompassing scientific, strategic, and operational dimensions (13, 17, 19).

To capture specific information on health malaria needs and contextual nuances, mixed-method baseline surveys were conducted between January and December 2022 and provided insights into the opportunities and challenges for community engagement.

## The involvement of community members as field workers

Curema alliance counts with the decisive contribution of DPAC Fronteira association as an intermediary between the community and scientific entities and with a team of Community Health Workers (CHWs) who underwent training in bi-directional learning spaces from February to March 2023 (18, 34). This training facilitated a productive exchange of ideas between CHWs and the coordination team, covering topics like the IEC strategy, community engagement techniques, and adapting tools and procedures for effective fieldwork. This joint work was very favorable for the launching of the intervention, which started gradually, in Suriname in March 2023 and on the Brazilian side in October 2023.

## Information, education, and communication tools

The Curema project meticulously designed its educational component from January 2022 to August 2023. The overall objective of the information, education and communication strategy is not only to improve malaria care behaviors; it also encompasses the promotion of community involvement. The starting point for this development were two qualitative surveys carried out by a social scientist among the study population during 2022, with the aim of exploring knowledge and representations concerning malaria, its symptoms and causes, and its treatment. Collaborative efforts involving CHWs, community members, researchers, and arts-based professionals led to the creation of accessible visual materials, through an iterative process, in consultation with members of the community or partner organizations as DPAC. The actual filmmaking for the project's video materials was carried out in Oiapoque in November 2022, with the direct participation of the community members involved in explaining the selected concepts to their peers with their own words. The IEC tools are available on the project's website (35) and presented detailed in Table 1.

**Table 1:** Tools and materials developed for the IEC component of the Curema intervention.

**Materials**	**Objectives**	**Audience**	**Communication channels**
Curema Spot video	- Present the project	The ASGM community	- Social networks - Through the CHW in informative meetings with the community. - TV of restaurants in the logistic sites. - Project APP
Video about malaria	- Present basic information about malaria. - Standardize the main messages to be explained.	The ASGM community and the Curema participants	- Social networks - Through the CHW in informative meetings with the community or during the training of the participants. - TV of restaurants in the logistic sites. - Project APP
Malaria explanation Sheets	- Present basic information about malaria. - Standardize the minimum information to be explained.	The ASGM community and the Curema participants	- Through the CHW in informative meetings with the community or during the training of the participants.
Malaria Game	- Deepen the explanations of malaria and enhance bi-directional communication.	The ASGM community and the Curema participants	- Through the CHW in informative meetings with the community or during the training of the participants.
Information note video	- Inform about all the aspects in line with the project information note.	Curema potential participants	- Through the CHW before proceeding with the inclusion of the participant prior to signing the informed consent form. - Project APP
Videos about how to use Malakit: RDT and treatment	- Explain how to use the Malakit.	Curema participants	- Social networks - Through the CHW during the training of the participants - Project APP
Malakit cover package	- Represent in a visual and clear way how the kit should be used	Curema participants	- Explanatory print on the plastic package of the kit to be attentively displayed with the CHW during the training and accessible to the participant upon use.
Radical cure brochure	- Represent in a visual and clear way how the radical cure treatment should be used.	Curema participants	- Explanatory print on the kit to be attentively displayed with the CHW during the training and accessible to the participant during treatment and follow-up.
Teach-Back cards	- Test the knowledge acquired by the participant	Curema participants	- Material to be used by the CHW to enhance dialogue and verification of knowledge acquired by the participant prior to the delivery of services: Malakit and/or radical cure.

The wide diversity of materials and the involvement of facilitators in their development provide a rich array of adapted options that can be selected based on specific situations and audiences. IEC tools, as infographics, videos, or games, were created to break down complex concepts and present information clearly and visually, making it accessible to people with limited literacy. The videos have been compressed to be sharable portable on individual mobile phones, most readily available for consultation when needed.

Some of the visual materials were adopted and adapted from Malakit project: several malaria explanation sheet, the Malakit illustrations, the rapid test performance guidance video, as well as the teach-back cards for the Malakit training.

The new materials developed in the context of the Curema project included short videos (Curema project's teaser, a video providing general information on malaria tailored to the local epidemiological context, a video explaining how to use the malakit treatment) featuring community members, CHWs, and researchers who explained their role to achieve malaria elimination.

Additionally, to adapt to the low literacy level of community participants the information note of the informed consent process was complemented by an adapted video format, easily understandable and repeatedly consultable by study participants. This video was scripted and performed by project's CHWs, as part of their training process. Its utilization was validated by the relevant Ethical Committees.

However, the most interactive educational item was the Malaria game, which offered the best access to understand the players' representations toward malaria and to explore the visual representation and sensory experience of specific matters of concern. The malaria game also proved to be a very useful material during CHWs training sessions.

The IEC strategies conveyed key messages to the community. It highlighted the feasibility of elimination without eradicating mosquitoes, emphasizing the importance of treating both symptomatic cases and those with hypnozoites *P. vivax* infections. The project raised awareness about ongoing malaria transmission, especially in gold mining areas, promoting collaborative efforts with the slogan “*Together we can eliminate malaria”*. It provided insights into the causative agent, symptoms, and the role of asymptomatic malaria in transmission. Stressing the curability of malaria through safe and appropriate treatment, the project emphasized compliance with drug regimens to prevent relapses, transmission, and antimalarial resistance.

The supporting IEC tools constitute an essential part of the mandatory training for participants included in the research who wish to receive at least one of the services offered by the intervention. Additionally, CHWs arrange communication meetings; conduct transect routes throughout the settlements to identify individuals on transit to gold mines; screen the videos at TV of restaurants; and use the project tablets and APP. Besides, CHWs actively engage with social media platforms, including Facebook and WhatsApp groups.

In addition, the overall project approach is adaptive, iterative, and continuously building. Thus, the communication between CHWs and coordinators of the study, as well as the supervision and monitoring exercise allowed the adaptation of the IEC strategy to anticipate and respond to context-specific needs that may arise throughout the ongoing project.

## A bottom-up outreach activity

The project capitalized on opportunities in the Brazilian territory and leveraging the DPAC Frontera association's social networking expertise. A 2-day health fair in Ilha Bela (conducted in November 2023), synchronized with the launch of the Curema intervention in this locality, addressed ASGM community expressed priorities. To develop this bottom-up outreach activity, DPAC Fronteira facilitated community consultations realized in August 2023 by a community health worker of the organization linked to the settlement's community, with the support of the project's social scientist. DPAC Fronteira subsequently raised awareness, mobilized and established partnerships with health and social local institutions linked to the municipal administration. These local actors have taken ownership of the initiative and made possible a multidisciplinary intervention to promote the health of this vulnerable population. The event included the Curema intervention, Chagas disease education, a cinema-debate on violence, social benefits guidance, discussions on abortion and women's rights, psychosocial advice, a beautician course for women's income generation, and children's activities. In consequence, Ilha Bela, often overlooked, gained attention, showcasing community involvement, actor's collaboration, and the attraction to Curema's presentation and to malaria elimination efforts.

## Discussion

Community engagement is considered an essential element for the success of health interventions. Whittaker and Smith (23) observed that historically, top-down approaches used for malaria control and eradication have not effectively utilized the potential of community engagement—a concept emphasized in the 1978 Alma Ata declaration on Primary Health Care. However, as regions progress toward malaria elimination, community engagement becomes increasingly challenging due to reduced disease risk perception (23, 27, 36–38).

Challenges for community engagement among mobile and hard-to-reach populations are not unique to the Guiana Shield but are also prevalent in various regions worldwide, such as Pacific Asia, the Greater Mekong, or the Amazon. These regions share common characteristics like remote locations, challenging environmental conditions, poor accessibility, and low population density. These factors affect ethnic minorities, refugees, displaced individuals, and groups engaged in activities like logging, mineral extraction, fishing, etc. (1, 39–41).

Smith and Whittaker (42) propose a shift from targeted interventions for mobile populations to a reactive geographical approach, addressing malaria hotspots. Mobility, they argue, is a multifaceted system interconnecting mobile and sedentary communities, with successful interventions documented using mobile clinics and workers (9, 15, 41).

On the other hand, implementing bottom-up health interventions based on community needs that amalgamate essential health services, education, access to clean water, and sanitation could promote community participation, and prove more relevant for underserved populations, while effectively managing targeted diseases as malaria (24, 36, 37, 43, 44). Despite recognizing the benefits, operationalizing such initiatives targeting ASGM communities in the Guiana Shield faces obstacles due to complex multilayered challenges (13, 14, 17, 19).

The Curema project adopts an adaptive, flexible approach rooted in a community-based research model, encouraging the information, consultation, and collaboration levels in the continuum of community engagement. Key elements include multidisciplinary collaboration, formative research, mixed evaluations, on-site visits, trust building, CHW training, and the IEC strategy. Decentralized malaria care initiatives with CHWs have successfully reduced malaria in remote areas (45) and are being implemented for radical treatment with G6PD testing in Cambodia (46).

Interventions targeting *P. vivax* malaria might face several challenges in terms of participation, because of a more demanding treatment regimen and silent hypnozoite carriage. The Curema project prerequisites such as G6PD testing, pregnancy testing, and clinical inquiries are necessary before administering the chloroquine + primaquine/tafenoquine treatment. Participants must be informed about the risks, educated to recognize potential severe side effects, and commit to follow-ups. These requirements emphasize the treatment's risks, potentially leading to negative risk-benefit perceptions among individuals. The therapeutic regimen stipulates 3 days of chloroquine combined with 7 days of primaquine, which might be challenging to adhere to, especially for asymptomatic individuals. Other studies also acknowledged limitations in the adherence in full regiment of Mass Drug Administration with Primaquine due to perceived and real fears of adverse drug effects, due to the duration of the regimen and limitations for following up (40, 46, 47). Therefore, the IEC strategy is crucial in facilitating a dialogue with the community, helping individuals weight the risks also understanding the potential benefits, at individual and collective scale. Future Curema project phases are expected to introduce a simpler regimen−3 days of chloroquine + a single dose of tafenoquine, which may enhance participation and adherence. The CUREMA project is currently ongoing, the implementation and the effectiveness of this complex intervention on malaria (and specifically *P. vivax*) transmission will be evaluated with a mixed-methods approach; main results are expected by the end of 2025.

To design effective IEC strategies and promote adherence, culturally sensitive, evidence-based education strategies are essential. These strategies should incorporate quantitative, qualitative, or mixed assessments, along with contextualized training for health workers (46, 48, 49). Curema IEC strategies were developed through iterative consultative and collaborative processes, formative research, and close supervision. There are also numerous experiences along literature on the co-design of educational tools in different formats to overcome low health literacy: print, media, radio, theater, community meetings, games, songs, social networks, app, etc. (43, 48, 50).

The Curema project facilitated the performance of health fair, a bottom-up outreach activity, in one of the most under-resourced settlements included in the project's activities. This experience suggests that vertical interventions for malaria elimination can serve as catalysts for bottom-up activities addressing other community health priorities. In addition, there is an opportunity to provide clear information on the health risks associated with gold mining. These outreach activities have the potential to foster community engagement, empowering individuals to take responsibility for their wellbeing, while also increasing awareness of malaria elimination efforts. These endeavors contribute to building a network between health and social care institutions and the community, promoting greater social cohesion. However, it is crucial to emphasize the involvement of key actors and the community's ownership of the initiative to ensure the sustainability of these actions.

To conclude, community participation is crucial to the success of health interventions, especially in malaria elimination plans. Top-down approaches to health intervention are not always sufficient to achieve substantial community participation, especially among underserved populations where adapted and practical innovations are required for highly complex contexts. The intervention of the Curema project was not in itself developed completely and spontaneously by the community, but rather was the shared initiative of researchers, health institutions and civil society organizations. Nevertheless, the community's engagement in the Curema project is a multi-faceted effort that is an essential element of the project. The experience of this project reinforces that of the earlier Malakit project: highly mobile populations, considered hidden and hard to reach, are in fact often easily accessible, ready for productive collaboration to promote their health and wellbeing in the broadest sense. The low literacy of these communities should not be a hindrance, but an opportunity to co-develop tools that are genuinely accessible and relevant. The tools developed in the context of the Curema project are by nature context specific. Nevertheless, this experience and the steps taken to produce them are an example that can be shared more widely, to all contexts facing similar challenges in the latest pockets of malaria transmission, to encourage and inspire community engagement efforts.

## Data Availability

The original contributions presented in the study are included in the article/supplementary material, further inquiries can be directed to the corresponding author.
